# Assessing pragmatic abilities of small children with and without ASD diagnosis

**DOI:** 10.1590/2317-1782/e20250083en

**Published:** 2026-03-17

**Authors:** Ingrid Ya I Sun, Thais Helena Ferreira Santos, Fernanda Dreux Miranda Fernandes

**Affiliations:** 1 Departamento de Fonoaudiologia, Fisioterapia e Terapia Ocupacional, Faculdade de Medicina, Universidade de São Paulo – USP - São Paulo (SP), Brasil.

**Keywords:** Language Development, Language Tests, Child Language, Autism Spectrum Disorder, Social Communication Disorder

## Abstract

**Purpose:**

DSM 5 indicates social communication deficit as one of the necessary criteria for the diagnosis of autism spectrum disorder. However, the pragmatic assessment of language is not as commonly considered as the formal aspects of language. The presented guide aims to consider communicative elements that include nonverbal aspects of language and the intentionality of communicative acts to identify the first signs of pragmatic alteration.

**Methods:**

The ROLPP (Observation Script of Language from a Pragmatic Perspective) is an observation script for socio-cognitive and pre-verbal language skills and communication necessary for language development preceding speech and part of the pragmatic language construct. The protocol was applied to children aged 18 to 36 months, consisting of 11 children with suspected ASD and discarded diagnosis, and 11 children with confirmed ASD diagnosis. A descriptive level of 5% (p <0.05) was considered statistically significant.

**Results:**

The results indicate that Participants with a confirmed diagnosis of ASD had higher scores than those in whom the diagnosis was discarded. Children with a confirmed diagnosis tended to score higher on the ROLPP overall (p <0.001), highlighting the impact of pragmatic language disorders in children with ASD, even at preschool age.

**Conclusion:**

These preliminary results indicate the possibility of identifying signs of disorders or pragmatic difficulties even before 36 months of age, with low application costs, including for use by professionals with little experience or contact with developmental disorders such as ASD.

## INTRODUCTION

The increasing incidence of Autism Spectrum Disorder (ASD) diagnoses^(1)^, driven by advances in scientific knowledge and widespread awareness, has raised important questions regarding early diagnosis and early intervention, as well as considerations about clinical intervention with increasingly younger children^(2,3)^. As is well established, the diagnosis of ASD is primarily clinical, and its signs emerge early in childhood, with varying levels of severity. In general, ASD can be diagnosed before 30 months of age. Recent studies suggest that symptom “stabilization” may be observed as early as 12 to 14 months of age^(4)^.

Despite this evidence, early diagnosis is still not consistently achieved in Brazil for several reasons. These include limited access to specialized care for large segments of the population, particularly those living in rural areas or with fewer socioeconomic resources — as well as public policy challenges related to the lack of reliable epidemiological data on this population^(5)^. The 2022 Brazilian Demographic Census, originally planned for 2020, was the first to include data collection on individuals with autism. However, this initiative was interrupted due to the COVID-19 pandemic. Preliminary census data indicate that Brazil’s population reached 203,062,512 individuals. In parallel, the Centers for Disease Control and Prevention (CDC) reported in 2023 an autism prevalence of approximately 1 in 36 children up to 8 years of age, corresponding to about 2.8% of the population^(1)^. Based on this estimate and the Brazilian population size, it is possible to infer that at least six million individuals in Brazil may be on the autism spectrum, considering that this prevalence rate accounts only for children up to 8 years of age^(6)^.

According to the Diagnostic and Statistical Manual of Mental Disorders, Fifth Edition (DSM-5), the diagnosis of Autism Spectrum Disorder is based on a set of behavioral and developmental criteria^(7)^. These criteria are organized into two main domains: persistent deficits in social communication and social interaction, and restricted, repetitive patterns of behavior, interests, or activities. Symptoms must be present from early development and must negatively impact autonomy and interpersonal relationships across multiple contexts.

The DSM-5 indicates that impairments in pragmatic aspects of language are intrinsic and present across the entire ASD population^(8,9)^. However, pragmatic language assessment is far less frequently considered than other formal language components. Evaluating functional language use and its variability across contexts is complex and challenging, often requiring specific training to observe, assess, and intervene in this domain^(10-12)^. As a result, functional language use, arguably the primary object of study in this population, is frequently neglected in therapeutic planning or described only qualitatively due to difficulties in measuring pragmatic behaviors. This limitation also hampers the establishment of pragmatic language goals in speech-language intervention.

The difficulties in social communication and language that are intrinsic to ASD underscore the essential role of speech-language pathologists in the assessment, diagnosis, and intervention processes for this population^(9,12)^. Pragmatic language skills, executive functions, and social cognition operate in an interdependent manner to enable individuals to express and understand their own intentions and perspectives, as well as those of others^(13)^. Therefore, these domains must be jointly considered during assessment and intervention. Pragmatics focuses on language use, not only on formal linguistic abilities, but also on the capacity to apply acquired skills across diverse communicative situations. This perspective requires consideration of the individual’s historical, social, cultural, political, and economic contexts in order to understand the quality of communication. Such an approach is essential in the assessment and follow-up of individuals with ASD^(14,15)^, as the primary deficit in this population lies not in the acquisition of formal language skills, but in their functional use. For example, a child with ASD who understands and verbally produces 100 words may still be unable to use those words functionally within an interaction^(16)^.

Assessing communicative functionality requires going beyond the mere identification or quantification of verbal language presence, particularly across different interactional contexts. Nevertheless, significant challenges remain in this type of evaluation, since one of the core assumptions of pragmatics is that language use in social contexts necessarily involves relationships between language users and their interlocutors^(17)^. Furthermore, speech-language assessments are often conducted within restricted or highly controlled interactional settings. Hyter^(14,18)^ highlights several foundational and critical points in pragmatic language assessment, emphasizing that assessment activities should be more authentic, functional, and qualitative in nature. It is also essential to consider communicative elements that include nonverbal aspects of language and the intentionality of communicative acts, focusing on skills that do not rely exclusively on verbal output.

Currently, the most widely used protocol in Brazil for pragmatic language assessment is the Functional Communication Profile, published as part of the ABFW – Child Language Test^(19)^. However, its application in private clinical settings is limited, largely due to its complexity and the need for specific professional training, as well as its restricted use in scientific research^(20)^.

Given these limitations, the present study aims to present preliminary performance results of children assessed with the Observation Script of Language from the Pragmatic Perspective (LOGPP)^(21)^, a pilot instrument developed as part of the first author’s doctoral research. The study also seeks to characterize and compare pragmatic language performance in children with and without a confirmed diagnosis of ASD using the LOGPP, with the goal of identifying pragmatic language alterations at an early stage, regardless of oral language abilities.

## METHODS

### The Protocol

The LOGPP (Language Observation Guide from the Pragmatic Perspective) is an observational tool designed to assess socio-cognitive and pre-verbal language skills that are essential for language development. These skills precede speech and are part of the pragmatic language construct. To define which skills should be observed and how they should be assessed, a comprehensive theoretical review was conducted as part of the first author’s doctoral research, which is summarized below.

Four areas of observation were defined in the LOGPP: Responsiveness, Eye Contact and Joint Attention, Socio-cognitive Aspects – Imitation and Symbolic Play.

#### 1. Responsiveness

All individuals with ASD, regardless of severity level, present pragmatic impairments and difficulties in contextual language comprehension^(22)^. These difficulties may be observed as increased response latency, inconsistent responding, when the child responds sometimes but not consistently, or even as an absence of response, such as not responding to their own name, which is a frequent concern reported by parents and caregivers of children with autism^(23)^.

From a pragmatic perspective, a more in-depth analysis reveals that reduced or absent responsiveness and engagement may be reflected in a partial or complete absence of communicative initiations^(3,4,8,16)^, thereby compromising interaction and communication.

Pragmatics is grounded in daily interactions among diverse groups of people who share worldviews and influences, and are influenced by their social environments and practices. These interactions are essential for learning and using social and cultural resources, as well as for developing knowledge that enables engagement across multiple contexts with a variety of interlocutors^(14)^. Observing and analyzing a child’s level of social engagement and responsiveness helps expand our understanding not only of the child’s interactional patterns, but also of their broader communicative context^(24,25)^.

#### 2. Development of Joint Attention

The development of Joint Attention (JA), which emerges during the first year of life, provides a neural experience that is fundamental for later social skill development^(26)^. Through this ability, individuals begin to appropriate the system of codes used in their sociocultural environment, sharing attention toward the same action, object, or situation^(27)^. Current research identifies JA as a predictor of intentionality, cognitive development, language acquisition, and social skills^(22,27,28)^.

Eye gaze is therefore a crucial aspect to be observed in young children, as it carries important indicators of joint attention, intentionality, and communication, particularly in children who do not yet communicate verbally^(29)^.

In the ASD population, joint attention is frequently identified as a core area of impairment and a key target for intervention. Several authors^(16,23,28,29)^ highlight that most children with ASD do not demonstrate collaborative or reciprocal engagement and rarely participate in symbolic or cultural activities. Studies indicate that children with ASD may present either an absence of joint attention or difficulties coordinating this skill during interactions^(22)^. Neuroimaging studies examining gaze-based joint attention tasks in individuals with ASD have also demonstrated atypical activation of neural systems associated with performance^(30,31)^.

Avoidance of eye contact, beyond being an early indicator, often persists into adulthood. Studies involving self-reports from autistic adults describe eye contact as triggering both emotional and physiological responses, frequently associated with feelings of intrusion, sensory overload, anxiety, and difficulty interpreting implicit social messages^(32,33)^.

#### 3. Socio-cognitive Aspects: Imitation

Imitation is a strong predictor of cognitive and language development. This skill emerges during the first year of life and develops rapidly in complexity throughout early childhood. Combined with other abilities, imitation enables children to construct shared symbols that are essential for language development. It is strongly influenced by social interaction and sustained through it^(34,35)^.

Imitation has been extensively studied and closely linked to intentionality, joint attention, social reciprocity, and communication^(3,36,37)^. These skills are considered fundamental for the later development of theory of mind and social communication^(34,37)^. In individuals with ASD, imitation difficulties are well documented^(34,35,38,39)^, whether in elicited or spontaneous imitation^(17)^.

In a study conducted by Pecukonis et al.^(40)^, the authors examined the relationships between expressive language and variables such as joint attention, imitation, and play in a sample of 37 minimally verbal children and adolescents with ASD. Their results demonstrated that imitation and play were significantly correlated with expressive language abilities.

#### 4. Socio-cognitive Aspects: Symbolic Play

Symbolic maturity is achieved through the ability to represent or meta-represent — that is, the capacity to symbolize concrete elements such as objects, animals, or people, as well as abstract elements such as situations, actions, and sensations^(24)^. This function involves earlier cognitive abilities, including deferred imitation and mental problem-solving skills, allowing the child to retain multiple forms of representation, as well as systems of signifiers and their associated meanings, which are necessary for language development^(41)^.

Early signs of symbolic play emerge prior to verbal language and play a critical role in enabling children to reach more complex linguistic levels^(42)^. Symbolic play is also closely intertwined with emotional, cognitive, social, and communicative domains^(43)^. Consequently, children with language disorders frequently present deficits in this area^(43,44)^, as do individuals with ASD^(45)^.

Numerous studies have demonstrated the positive effects of play-based interventions on the promotion of linguistic abilities^(45)^. Additionally, evidence suggests that children with ASD often experience challenges in acquiring symbolic play skills and in adapting these skills across different contexts^(45-47)^.

### Structure and Scoring of the LOGPP

The LOGPP was developed in a flowchart format to support clinical reasoning, allowing speech-language pathologists, even those without specific training in pragmatics, to identify different levels of skill development. The protocol is applied during a playful interaction, in which the clinician is instructed to observe the child’s performance in each target skill.

Based on the child’s observed performance, the clinician is guided to elicit either more or less complex manifestations of the same skill, facilitating identification of the child’s current developmental level. All interactions are video recorded to allow for scoring and later analysis, ensuring observation of the child’s best possible interactional performance.

Scoring is based on the level of complexity achieved for each skill. For each observed behavior, two outcomes are possible: presence or absence. When a skill is present and expected for the child’s developmental level, no points are assigned and the protocol directs observation toward a more complex skill. When a skill is absent, points are assigned and the protocol directs observation toward a less complex, earlier-developing skill. Thus, higher scores indicate greater impairment or a stronger warning sign of pragmatic language difficulties.

As its name suggests, the LOGPP is an observational script and does not aim to classify or diagnose typical development or disorder. Rather, it supports the construction of clinical reasoning in speech-language pathology by integrating pragmatic and functional language aspects with other domains relevant to language assessment.

### Procedures

The LOGPP was administered by the first author to 22 children aged 18 to 36 months, regardless of their primary communicative modality (verbal or nonverbal).

The study was approved by the Research Ethics Committee (REC) under protocol number 2.786.216. All caregivers provided informed consent, either in person or virtually via Google Forms.

Participants were divided into two groups. Group 1 (G1) consisted of 11 children with suspected ASD whose diagnosis was subsequently ruled out. Group 2 (G2) consisted of 11 children with suspected ASD and a confirmed diagnosis.

Inclusion criteria for G1 were:

- Age between 18 and 36 months;
- Suspected ASD based on clinical observation or a score above 3 on the M-CHAT total score or critical items;
- ASD diagnosis subsequently ruled out.

Inclusion criteria for G2 were:

- Age between 18 and 36 months;
- Suspected ASD based on clinical observation or a score above 3 on the M-CHAT total score or critical items;
- Confirmed ASD diagnosis based on specialized medical and speech-language evaluations.

The LOGPP application consists of observing skills across four areas that typically emerge during interaction. Prior to administration, the examiner familiarized herself with the protocol to ensure that situations eliciting each target skill were adequately incorporated into the interaction. All data collection involved dyadic interactions between the examiner (the first author) and the child, recorded using a mobile phone camera and later analyzed for scoring purposes.

All recordings were conducted in familiar environments. In this study, 100% of the recordings took place in speech-language pathology clinics where the children were already receiving assessment or intervention. Although the examiner was not a familiar interlocutor to the child, the session only began once the child appeared comfortable in the interaction. Parents and clinicians were invited to remain in the room but did not interfere with the procedure. The average duration of each interaction ranged from 15 to 20 minutes, depending on the child’s level of engagement.

Children were classified as verbal or nonverbal. Verbal children were defined as those producing consistent oral speech with appropriate semantic content, regardless of vocabulary size, with at least 75% intelligibility and phonetic-phonological patterns compatible with Brazilian Portuguese. Nonverbal children were defined as those with no oral productions demonstrating semantic or phonetic-phonological adequacy.

All video recordings were analyzed retrospectively. Analyses were conducted blindly, without knowledge of whether the ASD diagnosis had been confirmed or ruled out, to minimize bias. Scores were assigned according to LOGPP criteria, entered into an Excel spreadsheet, and subjected to statistical analysis.

Descriptive data analyses included absolute (n) and relative (%) frequencies, measures of central tendency (mean and median), and dispersion (standard deviation, minimum, and maximum values). Group comparisons for qualitative variables, including sex and verbal versus nonverbal status, were performed using the chi-square test.

Comparisons between study groups, adjusted for verbal and nonverbal status, were conducted using the Games–Howell test. A significance level of 5% (p < 0.05) was adopted. Data were analyzed using SPSS version 23 for Windows.

### Materials

The following materials were used for LOGPP data collection:

- A mobile phone camera to record adult–child interactions;
- Objects and toys of interest to the child.

When a child demonstrated restricted or specific interests, these items were incorporated into the assessment to facilitate engagement (e.g., using a preferred toy car during the Symbolic Play subtest, or a preferred object during the Responsiveness, Imitation, or Symbolic Play subtests).

## RESULTS

The sample consisted of 11 children with suspected ASD whose diagnosis was ruled out and 11 children with a confirmed diagnosis of ASD.

Table 1 presents the description of participants in the group with suspected ASD and a discarded diagnosis, including sex, communicative modality (CM), and age. The mean age of Group 1 was 2 years and 3 months.

**Table 1:** Description of subjects with ASD diagnosis ruled out

**Suspected ASD, ruled out**			
**Subj**	Sex	CM	Age
**1**	M	NV	1y9m
**2**	M	V	2y4m
**3**	M	NV	3y
**4**	M	NV	1y8m
**5**	M	V	2y4m
**6**	M	V	1y8m
**7**	F	NV	2y11m
**8**	F	NV	2y1m
**9**	F	V	3y
**10**	M	NV	1y8m
**11**	M	V	2y9m

**Caption:** ASD = Autism Spectrum Disorder; M = Male; F = Female; CM = Communicative Modality; V = Verbal; NV = Nonverbal; y = years; m = months

Table 2 presents the description of participants in the group with suspected ASD and a confirmed diagnosis, including sex, communicative modality, and age. The mean age of Group 2 was 2 years and 6 months.

**Table 2:** Description of subjects with ASD diagnosis confirmed

**Suspected ASD, confirmed**			
**Subj**	Sex	CM	Age
**1**	F	NV	2y9m
**2**	F	V	2y
**3**	M	NV	2y10m
**4**	M	NV	2y3m
**5**	M	V	2y7m
**6**	M	V	2y3m
**7**	M	V	2y7m
**8**	M	V	2y9m
**9**	F	V	3y
**10**	M	V	2y2m
**11**	M	NV	2y3m

**Caption:** ASD = Autism Spectrum Disorder; M = Male; F = Female; CM = Communicative Modality; V = Verbal; NV = Nonverbal; y = years; m = months

Table 3 shows a statistically significant association between communicative modality (verbal vs. nonverbal) and study group outcome. Children in the group with suspected ASD and a discarded diagnosis showed a higher proportion of nonverbal children (p < 0.001).

**Table 3:** Association between the variables of sex and verbal and nonverbal child outcomes in the study group

Variables		GROUP								Total		p^*^
				Suspected ASD, ruled out		Suspected ASD, confirmed						
				n	%	n	%			n	%	
SEX	Male			8	72.7	8	72.7			16	72.7	0.675
	Female			3	27.3	3	27.3			6	27.2	
												
Child	verbal			5	45.5	7	63.6			12	54.5	<0.001
	nonverbal			6	54.5	4	36.4			25	45.5	
												
Total				11	100	11	100			22	100.0	

* Chi-square

Tables 4 and 5 present the descriptive analyses of performance across the four LOGPP domains for the two study groups. Significant differences were observed between the group with suspected ASD and a discarded diagnosis and the group with suspected ASD and a confirmed diagnosis.

**Table 4:** Descriptive analysis by performance in the areas of the group with diagnosis ruled out

Ruled out	Responsiveness	JA/EC	Imitation	Symbolic Play	Total Score
Mean	18	10.5	8	16	52.5
Median	15	12.5	7.5	15	45
Standard Deviation	8.56	4.97	7.15	3.94	16.71
Minimum	10	5	0	10	35
Maximum	30	15	20	25	85

**Caption:** JA/EC = Joint Attention and Eye Contact

**Table 5:** Descriptive analysis by performance in the areas of the group with diagnosis confirmed

Confirmed	Responsiveness	JA/EC	Imitation	Symbolic Play	Total Score
Mean	30.91	34.55	16.82	23.18	105.45
Median	40	20	25	105	105
Standard Deviation	3.02	9.61	8.15	7.17	15.72
Minimum	25	15	5	10	80
Maximum	35	40	25	30	125

**Caption:** JA/EC = Joint Attention and Eye Contact

Overall, participants with a confirmed ASD diagnosis demonstrated higher scores than those whose diagnosis was ruled out. When comparing group means (Chart 1), statistically significant differences were observed across all four domains: Responsiveness (p = 0.009), Eye Contact and Joint Attention (p < 0.001), Imitation (p = 0.01), and Symbolic Play (p = 0.01). These findings indicate that children with a confirmed ASD diagnosis tend to present higher overall LOGPP scores (p < 0.001), highlighting the impact of pragmatic language impairments in children with ASD, even at preschool age.

**Chart 1:** Statistical analysis: comparison between groups

	**Responsiveness**	**JA/EC**	**Imitation**	**Symbolic Play**		**Total Score**
**P-value**	0.0009^*^	<0.0001*	0.01*	0.01*		<0.001*

* Significant difference considering p<0,05

**Caption:** JA/EC = Joint Attention and Eye Contact

## DISCUSSION

Based on the analyses conducted, the LOGPP proved to be a low-cost, easy-to-administer instrument that provides relevant information to be integrated into a comprehensive speech-language assessment battery for children with pragmatic language deficits.

Pragmatic impairment can be observed across a range of language and neurodevelopmental disorders, with symptoms exerting varying levels of impact on communication^(9)^. As important as the structural components of language are the individual’s ability to use these components across different contexts, situations, and communicative purposes. In cases of ASD, pragmatic impairments are present in all individuals^(8,9,11,12,46)^ and tend to persist throughout the lifespan^(7)^. Nevertheless, pragmatic deficits are frequently overlooked during assessment and, consequently, during intervention planning. In some individuals with ASD, evaluations focused on formal language domains—such as syntax or phonology—may be insufficient^(12,16)^, particularly for nonverbal children.

Analysis of group performance across the four LOGPP domains demonstrated that the group with a confirmed ASD diagnosis showed higher scores, indicating greater impairment, in all areas assessed (Responsiveness, Eye Contact and Joint Attention, Imitation, and Symbolic Play). These domains are strongly associated with pragmatic language abilities, reinforcing the relevance of assessing functional communication beyond formal linguistic skills.

A proportional analysis revealed a greater discrepancy between groups in the domains of Joint Attention (JA) and Eye Contact (EC), and a smaller discrepancy in Symbolic Play. This finding is noteworthy, given that JA and EC emerge earlier in development than Symbolic Play^(26,41,47)^. One possible explanation is that Responsiveness and JA/EC are interaction-based skills—present and required during social exchanges—whereas Imitation and Symbolic Play are more socio-cognitive skills. Although these abilities influence and depend on interaction, they may also occur independently. For instance, a child may imitate actions or engage in symbolic play without necessarily participating in reciprocal interaction. From this perspective, the four domains assessed by the LOGPP may be conceptualized as two broader clusters: Responsiveness and JA/EC on one hand, and Imitation and Symbolic Play on the other.

A study comparing gaze fixation toward faces in children with and without ASD found no differences between groups in fixation time on the eye region. However, when an external distractor competing with the face, such as geometric shapes was introduced, group differences became more pronounced^(48)^. This finding suggests that children with ASD may respond to social stimuli, albeit in atypical or inefficient ways. A similar interpretation applies to pragmatic language analysis: examining a single behavior or skill in isolation may not necessarily indicate impairment, whereas analyzing how that behavior is used functionally across contexts allows for a clearer understanding of social communication deficits.

Consistent with the present findings, when verbal and nonverbal status was considered, nonverbal children demonstrated higher mean and median scores across the assessed domains (p < 0.05), indicating greater difficulties. Although the LOGPP does not depend on verbal output for administration, it may serve as an early indicator of language development delays by capturing pragmatic language deficits rather than focusing solely on the presence or absence of speech. In early childhood, distinguishing between primary social communication deficits and secondary effects related to broader language, cognitive, social, emotional, or motor development remains particularly challenging. During this initial developmental period, these domains are highly interdependent and continuously evolving, further complicating early diagnosis^(49,50)^.

Regarding the effectiveness of the protocol, the LOGPP demonstrated the ability to identify impairments across four developmental domains that are essential for language acquisition: responsiveness, eye contact and joint attention, imitation, and symbolic play. These findings were expected, as deficits in social communication represent a core characteristic of ASD. Comparisons between group means revealed statistically significant differences across all four domains (Responsiveness p = 0.009; Eye Contact and Joint Attention p < 0.001; Imitation p = 0.01; Symbolic Play p = 0.01). These results indicate that children with a confirmed ASD diagnosis tend to obtain higher overall LOGPP scores (p < 0.001), underscoring the impact of pragmatic language impairments in children with ASD, even at very young ages.

## CONCLUSION

These preliminary LOGPP findings indicate the possibility of identifying signs of pragmatic language disorders or difficulties before 36 months of age. The protocol may be used by speech-language pathologists with limited experience or minimal prior contact with neurodevelopmental disorders such as ASD, supporting earlier screening and referral for specialized assessment.

The LOGPP proved to be a low-cost and effective resource that is easy to implement in clinical practice. Nevertheless, further studies are needed to better understand its strengths and limitations. It is also important to consider the relevance of systematic observation models that focus on children’s spontaneous communication with familiar adults, minimizing the influence of motivational, familiarity-related, linguistic, and cultural factors.

Given that early assessment and intervention are among the most influential factors in the prognosis of children with language impairments, the use of instruments that facilitate and streamline the initial stages of this process is essential.
