# Supplementary material for: Assessing pragmatic abilities of small children with and without ASD diagnosis
Source: Codas. 2026 Mar 17;38(2):e20250083. doi: 10.1590/2317-1782/e20250083en (PMC13142163; doi:10.1590/2317-1782/e20250083en)
Supplement: Translation

# Translation: Avaliação de habilidades pragmáticas de crianças pequenas com e sem diagnóstico de TEA

**DOI:** 10.1590/2317-1782/e20250083pt

**Authors:** Ingrid Ya I Sun, Thais Helena Ferreira Santos, Fernanda Dreux Miranda Fernandes

**Affiliations:** 1 Departamento de Fonoaudiologia, Fisioterapia e Terapia Ocupacional, Faculdade de Medicina, Universidade de São Paulo – USP - São Paulo (SP), Brasil.

**Keywords:** Desenvolvimento da Linguagem, Testes de Linguagem, Linguagem Infantil, Transtorno do Espectro Autista, Transtorno de Comunicação Social

## Abstract

**Objetivo:**

O DSM 5 indica o déficit de comunicação social como um dos critérios necessários para o diagnóstico do transtorno do espectro autista. No entanto, a avaliação pragmática da linguagem não é tão comumente considerada quanto os aspectos formais da linguagem. O roteiro apresentado visa considerar elementos comunicativos que incluem aspectos não verbais da linguagem e a intencionalidade dos atos comunicativos para identificar os primeiros sinais de alteração pragmática.

**Método:**

O Roteiro de Observação da Linguagem na Perspectiva Pragmática (ROLPP) é um roteiro de observação de habilidades sociocognitivas e pré-verbais necessárias para o desenvolvimento da linguagem que precedem a fala. Tais habilidades fazem parte do construto da linguagem pragmática. O protocolo foi aplicado em crianças de 18 a 36 meses, sendo composto por 11 crianças com suspeita de TEA e diagnóstico descartado, e 11 crianças com diagnóstico confirmado de TEA. Um nível descritivo de 5% (p < 0,05) foi considerado estatisticamente significativo.

**Resultados:**

Os resultados indicam que os participantes com diagnóstico confirmado de TEA apresentaram pontuações mais altas do que aqueles em que o diagnóstico foi descartado. Crianças com diagnóstico confirmado tenderam a pontuar mais alto no ROLPP geral (p < 0,001), destacando o impacto dos transtornos pragmáticos de linguagem em crianças com TEA, mesmo em idade pré-escolar.

**Conclusão:**

Esses resultados preliminares indicam a possibilidade de identificar sinais de transtornos ou dificuldades pragmáticas mesmo antes dos 36 meses de idade, com baixos custos de aplicação, inclusive para uso por profissionais com pouca experiência ou contato com transtornos do neurodesenvolvimento como o TEA.

## INTRODUÇÃO

O crescente aumento da incidência do diagnóstico de Transtornos do Espectro do Autismo (TEA)^(1)^ graças ao avanço da ciência e da conscientização massiva, tem levantado questões voltadas ao diagnóstico e intervenção precoces e considerações quanto à intervenção com crianças cada vez mais novas na prática clínica^(2,3)^. Como se sabe, o diagnóstico de TEA é, principalmente clínico, e os sinais surgem logo na primeira infância, em maior ou menor grau de severidade. Em geral, é possível diagnosticar o TEA antes dos 30 meses de idade. Estudos recentes têm sugerido que é possível observar a “estabilização” dos sintomas por volta dos 12 a 14 meses de idade^(4)^. O diagnóstico precoce ainda não ocorre de forma consistente no Brasil por variados motivos. Tanto por falta de acesso ao atendimento especializado para toda a população, principalmente rural e com menos recursos, como por questões de políticas públicas que envolvem a falta de dados sobre essa população no Censo^(5)^. A edição de 2022 do IBGE, planejada para ser realizada em 2020, foi a primeira edição que considerou levantar os dados da população com autismo. No entanto, tal plano foi interrompido devido à pandemia de COVID-19. Os dados preliminares do Censo mostram que a população do Brasil atingiu 203.062.512 pessoas. E o CDC (Centers for Disease Control and Prevention) divulgou em 2023 que a prevalência de autismo seria em torno de 1 em cada 36 crianças de até 8 anos, cerca de 2,8% da população^(1)^. Considerando essa estatística e a população brasileira de 203 milhões, pode-se estimar pelo menos 6 milhões de pessoas com TEA no Brasil, já que tal informação considera apenas crianças até 8 anos^(6)^.

O diagnóstico do Transtorno do Espectro Autista (TEA) segundo Manual Diagnóstico e Estatístico de Transtornos Mentais, 5ª edição (DSM-5) é baseado em uma série de critérios comportamentais e de desenvolvimento^(7)^. Os critérios estão organizados em duas áreas principais: déficits persistentes na comunicação social e interação social e padrões repetitivos e restritos de comportamentos, interesses ou atividades.

Os sintomas devem ser observados desde o início do desenvolvimento e ter um impacto negativo na autonomia e nas relações interpessoais em diversas áreas. O DSM 5^(7)^ indica prejuízo da linguagem nos aspectos pragmáticos é presente e intrínseco a todos dessa população^(8,9)^. Mas a avaliação da linguagem pragmática não é tão comumente considerada como outros aspectos de linguagem formal. Considerar os aspectos funcionais e as suas variações diante de diferentes contextos é desafiador e complexo, exigindo muitas vezes um treinamento para olhar, avaliar e intervir nessa área^(10-12).^. Dessa forma, o uso funcional da linguagem, que deveria ser o objeto de estudo principal da linguagem desses indivíduos, é frequentemente negligenciado e considerado como secundário no plano terapêutico ou apenas descrito de forma qualitativa pela dificuldade de obter e mensurar esses dados, consequentemente tornando difícil também estabelecer objetivos de linguagem pragmática na intervenção fonoaudiológica.

A dificuldade de comunicação social e linguagem, intrínseca na população com TEA, sugere que o fonoaudiólogo é um dos profissionais essenciais na avaliação, no diagnóstico e intervenção dirigida a essa população^(9,12)^. As habilidades de linguagem pragmática, funções executivas e cognição social trabalham de forma interdependente para expressarmos e compreendermos as nossas intenções e perspectivas e as do outro^(13)^, sendo de extrema importância considerá-las na avaliação e intervenção. A pragmática estuda o uso da linguagem, ou seja, a compreensão para além das habilidades em aspectos formais de linguagem, mas também na capacidade de usar as habilidades adquiridas em variadas situações de comunicação. Para isso é necessário considerar o contexto histórico, social, cultural, político e econômico do indivíduo avaliado, para compreender a qualidade da comunicação. Esse olhar é imprescindível na avaliação e no acompanhamento fonoaudiológico de indivíduos com TEA^(14,15)^, já que o maior déficit desses indivíduos não está na aquisição de habilidades formais, mas sim no uso dessas habilidades. Uma criança com TEA que compreende e expressa verbalmente 100 vocábulos pode não conseguir usar funcionalmente tais vocábulos durante uma interação, por exemplo^(16)^.

Uma forma de analisar a funcionalidade da comunicação é ir além de identificar e quantificar a presença ou ausência da linguagem verbal, principalmente em contextos diferentes de interação. Ainda assim, temos muitos desafios nesse tipo de avaliação, já que uma das premissas da pragmática é de que o uso da linguagem em contextos sociais exige necessariamente a relação entre os usuários da língua e seus interlocutores^(17).^ Além disso, o contexto da avaliação fonoaudiológica costuma ser ainda muito pautado em uma interação restrita ou dentro de um contexto específico. Hyter^(14,18)^ sugere alguns pontos críticos e basais na hora de avaliar a linguagem pragmática. A autora enfatiza que as atividades de avaliação utilizadas devem ser mais autênticas, mais funcionais e mais qualitativas. É válido considerar também os elementos comunicativos que incluem aspectos não-verbais da linguagem e a intencionalidade dos atos comunicativos, observando habilidades que não dependam apenas da verbalização ou oralidade do indivíduo.

O protocolo mais utilizado atualmente no Brasil para avaliar a linguagem pragmática é o Perfil Funcional da Comunicação, publicado no ABFW-Teste de Linguagem Infantil^(19)^. No entanto, a sua aplicação é bastante reduzida em clínicas privadas por fonoaudiólogos que obtiveram treinamento específico e em pesquisas científicas devido à sua complexidade^(20)^.

Diante disso, o presente estudo tem como objetivo apresentar alguns resultados do desempenho de crianças no Roteiro de Observação de Linguagem na Perspectiva Pragmática (ROLPP)^(21)^, estudo piloto desenvolvido em uma pesquisa de doutorado pela primeira autora; caracterizar e comparar o desempenho em linguagem na perspectiva pragmática de crianças com e sem diagnóstico de TEA através do ROLPP, visando identificar precocemente as alterações pragmáticas de linguagem, independente da oralidade.

## MÉTODO

### O protocolo

O ROLPP (Roteiro de Observação de Linguagem na Perspectiva Pragmática) é um roteiro de observação de habilidades de linguagem sócio-cognitivas e pré-verbais necessárias para o desenvolvimento de linguagem. Essas habilidades precedem a fala e fazem parte do constructo de linguagem na perspectiva pragmática. Para determinar quais as habilidades e como observá-las, foi realizado um levantamento teórico no estudo de doutorado e sintetizado a seguir. Foram definidas quatro (4) áreas de observação nesse roteiro: Responsividade, Contato Ocular e Atenção Compartilhada, Aspectos sócio-cognitivos – Imitação e Aspectos sócio-cognitivos – Jogo Simbólico.

#### Responsividade

Todos os indivíduos com TEA, independente do grau de severidade, apresentam alterações pragmáticas e de compreensão contextual de linguagem^(22)^. Essa dificuldade pode ser observada na maior latência de tempo ou inconsistência na resposta, ou seja, a criança responde, mas não sempre. Ou até na ausência de resposta para o próprio nome, o que é frequentemente uma queixa dos pais de cuidadores de crianças com autismo^(23)^.

Na perspectiva pragmática, em uma análise mais aprofundada, a falta ou a ausência de responsividade ou engajamento do indivíduo pode ser observada pela ausência completa ou parcial de iniciativas comunicativas^(3,4,8,16)^, comprometendo assim a interação e a comunicação desses indivíduos.

A pragmática consiste em interações diárias de um determinado grupo variado de pessoas, compartilhando visões de mundo, influenciando e sendo influenciado pelo meio em que vivem e suas práticas sociais. Essas interações são essenciais para aprender e usar recursos sociais e culturais, desenvolvendo conhecimento para se envolver em vários contextos com uma variedade de interlocutores^(14)^. Observar e analisar o nível de engajamento social/responsividade da criança nos ajuda a expandir o entendimento sobre a forma e o ambiente de interação não apenas da criança avaliada, mas também de seu contexto comunicativo^(24,25)^.

#### Desenvolvimento de Atenção Compartilhada

O desenvolvimento da Atenção Compartilhada (AC) que ocorre no primeiro ano de vida da criança permite uma experiência cerebral que é fundamental para o desenvolvimento de habilidades sociais no futuro^(26)^. É a partir dessa habilidade que o indivíduo começa a se apropriar do sistema de códigos utilizados em seu meio sociocultural, dividindo a mesma atenção para a mesma ação, objeto ou situação^(27)^. Atualmente, estudos já indicam a habilidade de AC como preditor da intencionalidade desenvolvimento cognitivo, de linguagem e habilidades sociais^(22,27,28)^.

O olhar, então, é indiscutivelmente um aspecto importante a ser observado em crianças pequenas por carregar em si indícios de atenção compartilhada, intencionalidade e comunicação, principalmente daqueles que ainda não se comunicam verbalmente^(29)^.

Para a população com TEA, a AC é frequentemente indicada como uma área imprescindível de alteração e intervenção. Autores^(16,23,28,29)^ ressaltam que a maioria das crianças com TEA não apresenta engajamento colaborativo ou recíproco, bem como raramente participa de atividades culturais e simbólicas. Estudos mostram que as crianças com TEA podem apresentar desde a ausência da AC como a dificuldade de coordenar tal habilidade durante as interações^(22)^. Outros estudos que focam no comportamento de indivíduos com TEA em tarefas de AC baseadas na direção do olhar em estudos de imagem, mostram alguma ativação atípica do sistema neural associado à performance^(30,31)^. O comportamento de evitar o olhar, além de ser um indicador precoce, é algo que persiste mesmo na vida adulta. Em estudos que consideram o relato de indivíduos adultos com TEA, eles referem que o contato ocular causa reações tanto emocionais como fisiológicas. Se sentem “invadidos” com frequência, com sobrecarga sensorial e até mesmo ansiedade, além da dificuldade de interpretar a mensagem social não explícita verbalmente^(32,33)^.

#### Aspectos sócio-cognitivos: imitação

A habilidade de imitação é um preditor do desenvolvimento cognitivo e de linguagem. Tal habilidade surge no primeiro ano de vida e se desenvolve de forma intensiva, quanto ao seu nível de complexidade, logo nos anos iniciais da vida da criança. É através dessa habilidade, combinada com outras, que as crianças constroem os símbolos compartilhados, essenciais para o desenvolvimento de linguagem. Tal habilidade é fortemente influenciada pela interação social e sustentada por ela^(34,35)^. A imitação vem sendo estudada e fortemente relacionada com a intencionalidade, atenção compartilhada, reciprocidade social e comunicação^(3,36,37)^. Essas habilidades são consideradas essenciais para o desenvolvimento da teoria da mente e da comunicação social no futuro^(34,37)^. Em indivíduos com TEA, sabe-se que a dificuldade para a imitação é evidente^(34,35,38,39)^, seja em imitação provocada ou espontânea^(17)^. Em um estudo conduzido por Pecukonis et al.^(40)^, eles buscaram observar as relações existentes entre a linguagem expressiva e um conjunto de variáveis como a atenção conjunta, a imitação e a brincadeira, em uma amostra de 37 crianças e adolescentes minimamente verbais com TEA. Os resultados revelaram que a imitação e a brincadeira estavam significativamente correlacionadas com a linguagem expressiva.

#### Aspectos sócio-cognitivos: jogo simbólico

A maturidade simbólica se dá pela habilidade de representar ou metarrepresentar, ou seja, a capacidade de simbolizar desde elementos concretos como objetos, animais ou pessoas, até elementos abstratos como situações, ações e sensações^(24)^. Tal função envolve habilidades cognitivas anteriores como a imitação diferida e capacidade de utilizar e resolver problemas mentais, podendo reter variadas formas de representação, assim como o sistema de significantes e a associação aos seus signos, que são necessários para o desenvolvimento de linguagem^(41)^. Os primeiros sinais da construção do jogo simbólico surgem antes da linguagem verbal e é um fator preponderante para que a criança alcance níveis mais complexos linguísticamente^(42)^, além da interrelação entre as áreas emocionais, cognitivas, sociais e de comunicação^(43)^. Dessa forma, é comum encontrarmos crianças com transtornos de linguagem com déficits nessa área^(43,44)^, assim como acontece em indivíduos com TEA^(45)^. Diversos estudos mostram os efeitos positivos das intervenções baseadas em brincadeiras simbólicas na promoção de capacidades linguísticas^(45)^. Além disso, há evidências de que as crianças com TEA enfrentam frequentemente desafios na aquisição e desenvolvimento de jogo simbólico e na adaptação dessa habilidade em diferentes contextos^(45-47)^.

O roteiro foi construído em formato de fluxograma para auxiliar o olhar do profissional fonoaudiólogo, mesmo sem treinamento em pragmática, para os diferentes níveis das habilidades. Toda a aplicação se dá em situação de interação lúdica na qual o profissional é instruído a observar o desempenho da criança na habilidade em questão. Dependendo do desempenho da criança, o profissional é direcionado a incitar habilidades mais ou menos complexas daquela habilidade, facilitando a compreensão do nível de aquisição em que a criança se encontra. Toda a interação é gravada para possível pontuação e análise posterior, visando promover e observar a melhor interação possível da criança. A pontuação se dá pelo nível de complexidade de cada habilidade. No fluxograma, para cada habilidade observada existem duas possibilidades. A presença ou a ausência da habilidade. Em casos de habilidade presente/esperada, a criança não pontua e o roteiro indica a observação de uma habilidade mais complexa. Já quando a habilidade em questão está ausente, a criança pontua e o roteiro indica a observação de uma habilidade anterior, menos complexa. Ou seja, quanto maior a pontuação, maior o comprometimento na área ou sinal de alerta para a alteração pragmática. O ROLPP, como o próprio nome diz, é um roteiro de observação e não tem o objetivo de classificar ou categorizar normalidade ou transtorno. Mas sim, auxiliar a construção do raciocínio clínico fonoaudiológico considerando os aspectos pragmáticos e funcionais de linguagem em conjunto com outros aspectos necessários na avaliação de linguagem.

### Procedimentos

O ROLPP foi aplicado pela primeira autora com 22 crianças entre 18 e 36 meses de idade independentemente do meio comunicativo predominante, ou seja, tanto em verbais como não verbais.

O estudo foi aprovado pelo Comitê de Ética em Pesquisa (CEP) sob parecer número 2.786.216. Todos os responsáveis assinaram o termo de consentimento livre e esclarecido presencialmente ou virtualmente (via google forms).

Neste estudo, o ROLPP foi aplicado em dois (2) grupos de crianças. O G1 com 11 crianças com suspeita de TEA, mas com diagnóstico descartado e o G2 com 11 crianças com suspeita de TEA e com diagnóstico confirmado.

Os critérios de inclusão para o G1 foram:

- Idade entre 18 meses e 36 meses
- Crianças com suspeita de TEA através de observação clínica ou pontuação acima de 3 no resultado ou em itens considerados críticos no M-CHAT.
- Crianças que tiveram a hipótese de TEA descartada.

Os critérios de inclusão para o G2 foram:

- Idade entre 18 meses e 36 meses
- Crianças com suspeita de TEA através de observação clínica ou pontuação acima de 3 no resultado ou em itens considerados críticos no M-CHAT
- Diagnóstico confirmado de TEA mediante a avaliação médica e fonoaudiológica especializada.

A aplicação do ROLPP consiste em um roteiro de observação de habilidades, divididas em quatro áreas, apresentadas comumente em uma interação. Para isso, foi necessário se familiarizar com o roteiro anteriormente à interação, para garantir que as situações que incitam determinadas habilidades fossem estimuladas na interação. Todas as coletas foram realizadas em interação dual entre a aplicadora (a primeira autora) e a criança, registradas por câmera de celular e analisadas posteriormente para a pontuação. Os registros foram realizados em ambientes familiares. No caso desse estudo, 100% dos registros foram realizados em ambientes familiares para as crianças, em consultórios fonoaudiológicos onde as crianças já estavam passando por avaliação ou acompanhamento. A avaliadora não era um interlocutor familiar para as crianças no momento da coleta, mas a aplicação era iniciada apenas depois que a criança se demonstrava à vontade na interação. Os pais e fonoaudiólogos eram convidados para permanecer na sala, mas não interferiram na aplicação. O tempo médio de interação e aplicação era de 15 a 20 minutos, variando conforme o engajamento da criança.

Todas as crianças foram classificadas como verbais ou não verbais. Foram consideradas verbais, crianças com produções orais consistentes e adequadas semanticamente, independentemente da quantidade de vocábulos, e com 75% de inteligibilidade e compatível com as regras formais da linguagem fonética-fonológica, do português brasileiro. Foram consideradas não verbais, as crianças com 0% de produções orais com adequação semântica e fonético-fonológica.

Todas as gravações foram posteriormente analisadas. As análises foram feitas de forma cega, sem que a avaliadora soubesse se o diagnóstico de TEA foi confirmado ou descartado para evitar viés. A pontuação foi realizada conforme os critérios do ROLPP, tabuladas em uma planilha Excel e tratadas estatisticamente.

Foi realizada a análise descritiva dos dados por meio de frequências absolutas (n) e relativas (%), medidas de tendência central (média e mediana) e dispersão (desvio-padrão, valores mínimo e máximo).

Na comparação entre os dois (2) grupos de estudo conforme variáveis qualitativas, sexo e criança verbal ou não verbal, foi aplicado o teste do Qui-quadrado.

A comparação entre os grupos de estudo, ajustada pelas variáveis crianças verbal e não verbal, foi realizada pelo teste de Games-Howell. Assumiu-se um nível descritivo de 5%(p<0.05) para significância estatística. Os dados foram digitados em planilha Excel e analisados no programa SPSS versão 23 para Windows.

### Materiais

Para a coleta de dados do ROLPP foram utilizados:

- Câmera de celular para gravar a interação adulto e criança
- Objetos e brinquedos de interesse da criança. Quando a criança apresentava um interesse restrito ou específico por algum objeto/brinquedo, o mesmo podia ser incluído na aplicação para ajudar no engajamento. Por exemplo: Inserir um carrinho de preferência no subteste de Jogo Simbólico ou um objeto de interesse no subteste de Responsividade, Imitação ou como elemento abstrato no Jogo Simbólico.
- Objetos ou brinquedos em miniatura para o subteste de jogo simbólico como, alimentos, carrinhos, bonecos etc.

## RESULTADOS

A amostra foi composta por 11 crianças com suspeita de TEA e com diagnóstico descartado, e 11 crianças com diagnóstico de TEA confirmado.

Na Tabela 1, segue a descrição de sexo, meio comunicativo (MC) e a idade. A média de idade do grupo 1 com suspeita de TEA, descartado foi 2 anos e 3 meses.

**Tabela 1:** Descrição de sujeitos com TEA descartado

**Suspeita de TEA, descartada**			
**Suj**	Sexo	MC	Idade
**1**	M	NV	1a9m
**2**	M	V	2a4m
**3**	M	NV	3a
**4**	M	NV	1a8m
**5**	M	V	2a4m
**6**	M	V	1a8m
**7**	F	NV	2a11m
**8**	F	NV	2a1m
**9**	F	V	3a
**10**	M	NV	1a8m
**11**	M	V	2a9m

**Legenda:** TEA = Transtorno do Espectro Autista; M = Masculino; F = Feminino; MC = Meio Comunicativo; V = Verbal; NV = Não Verbal; a = anos; m = meses

Na Tabela 2, a descrição de sexo, MC e a idade. A média de idade do grupo 2 com suspeita de TEA, confirmado foi 2 anos e 6 meses.

**Tabela 2:** Descrição de sujeitos com TEA confirmado

**Suspeita de TEA, confirmada**			
**Suj**	Sexo	MC	Idade
**1**	F	NV	2a9m
**2**	F	V	2a
**3**	M	NV	2a10m
**4**	M	NV	2a3m
**5**	M	V	2a7m
**6**	M	V	2a3m
**7**	M	V	2a7m
**8**	M	V	2a9m
**9**	F	V	3a
**10**	M	V	2a2m
**11**	M	NV	2a3m

**Legenda:** TEA = Transtorno do Espectro Autista; M = Masculino; F = Feminino; MC = Meio Comunicativo; V = Verbal; NV = Não Verbal; a = anos; m = meses

Na Tabela 3 houve associação estatisticamente significativa entre a variável criança verbal e não verbal ao desfecho. Verifica-se que o grupo de crianças com “suspeita de TEA, descartado”, apresentaram maior percentual de crianças não verbais (p<0.001).

**Tabela 3:** Associação entre as variáveis sexo e criança verbal e não verbal ao desfecho grupo de estudo

Variáveis		GRUPO								Total		p^*^
		Suspeita de TEA, descartado				Suspeita de TEA, confirmado						
				n	%	n	%			n	%	
SEXO	Masculino			8	72,7	8	72,7			16	72,7	0,675
	Feminino			3	27,3	3	27,3			6	27,2	
												
Criança	Verbal			5	45,5	7	63,6			12	54,5	<0,001
	Não verbal			6	54,5	4	36,4			25	45,5	
												
Total				11	100	11	100			22	100,0	

* Qui-quadrado

Na Tabela 4 e 5, verifica-se que “suspeita de TEA, descartada” foi diferente significativamente do grupo “suspeita de TEA, confirmada”.

**Tabela 4:** Análise descritiva por desempenho nas áreas do grupo com diagnóstico descartado

Descartado	Responsividade	CO e AC	Imitação	Jogo simbólico	Score total
Média	18	10,5	8	16	52,5
Mediana	15	12,5	7,5	15	45
Desvio padrão	8,56	4,97	7,15	3,94	16,71
Mínimo	10	5	0	10	35
Máximo	30	15	20	25	85

**Legenda:** Co e AC = Contato Ocular e Atenção Compartilhada

**Tabela 5:** Análise descritiva por desempenho nas áreas do grupo com diagnóstico confirmado

Confirmado	Responsividade	CO e AC	Imitação	Jogo simbólico	Score total
Média	30,91	34,55	16,82	23,18	105,45
Mediana	40	20	25	105	105
Desvio Padrão	3,02	9,61	8,15	7,17	15,72
Mínimo	25	15	5	10	80
Máximo	35	40	25	30	125

**Legenda:** Co e AC = Contato Ocular e Atenção Compartilhada

É possível observar nos resultados que os participantes com diagnóstico confirmado de TEA apresentaram escores mais elevados do que os participantes para os quais o diagnóstico foi descartado. Ao comparar as médias dos dois grupos (Quadro 1) notou-se que houve diferença significativa comparando as quatro áreas (valores de p: Responsividade 0,009; Contato Ocular e Atenção Compartilhada <0,001; Imitação 0,01; Jogo Simbólico 0,01). Isso mostra que crianças com diagnóstico confirmado tendem a ter maior pontuação no ROLPP em geral (p-valor: <0,001), evidenciando os impactos das alterações de linguagem pragmática em crianças com TEA mesmo em idade pré-escolar.

**Quadro 1:** - Análise estatística: Comparação entre grupos

	**Responsividade**	**CO e AC**	**Imitação**	**Jogo Simbólico**	**Score total**
**P-valor**	0,0009^*^	<0,0001*	0,01*	0,01*	<0,001*

* Diferença significativa considerando p<0,05

**Legenda:** Co e AC = Contato Ocular e Atenção Compartilhada

## DISCUSSÃO

A partir da análise realizada, é possível afirmar que o ROLPP se mostrou um instrumento de fácil aplicação e baixo custo, que fornece informações importantes para integrar uma bateria de avaliação fonoaudiológica de crianças com déficits de linguagem pragmática.

O déficit pragmático pode ser observado em diversos transtornos de linguagem e neurodesenvolvimento, e os sintomas têm diferentes níveis de impacto na comunicação^(9)^. Tão importante quanto os componentes da estrutura da linguagem é a capacidade de utilizar esses componentes em diferentes contextos, situações e propósitos. Nos casos de TEA, os prejuízos nas habilidades pragmáticas estão presentes em todos^(8,9,11,12,46)^ além de ser persistente ao longo da vida^(7)^. No entanto, os sintomas da deficiência na pragmática são frequentemente preteridos e negligenciados durante a avaliação e assim permanecem durante a intervenção. E a avaliação de habilidades estruturais da linguagem, como áreas de sintaxe ou fonologia, em alguns indivíduos com TEA^(12,16)^ podem não ser suficientes, além de não serem possíveis em crianças não verbais, por exemplo. Observando o desempenho dos grupos nas quatro áreas, é possível afirmar que o grupo com o diagnóstico de TEA confirmado apresentou escores mais alto, ou seja, maior defasagem em todas as áreas (Responsividade, CO e AC, Imitação e Jogo Simbólico) que possuem uma forte correlação com a habilidade pragmática da linguagem.

Ao fazermos uma análise de proporção, existe uma discrepância maior entre os grupos na área de Atenção Compartilhada (AC) e Contato Ocular (CO) e menor em Jogo Simbólico (JS). O que é interessante, já que a habilidade de AC e CO é anterior à habilidade de JS no desenvolvimento infantil^(26,41,47)^.

Uma das hipóteses possíveis é que a Responsividade e AC e CO são habilidades inerentes da interação, presentes, necessárias durante a troca. Já a Imitação e JS são habilidades mais sócio-cognitivas. Apesar de serem habilidades que dependem e influenciam a interação, são habilidades que podem ocorrer de forma independente uma da outra. Por exemplo, uma criança pode imitar e realizar um jogo simbólico sem precisar interagir, de fato. Sob essa ótica, podemos subdividir as quatro áreas em duas, sendo Responsividade e AC e CO de um lado e Imitação e Jogo simbólico do outro.

Um estudo^(48)^ comparou a fixação do olhar para rostos entre crianças com e sem TEA. E observaram que crianças com TEA não diferem das crianças sem TEA no tempo de fixação na região dos olhos. No entanto, quando introduziram um distrator externo em competição ao rosto, que no caso foram formatos geométricos, a diferença entre os grupos é maior. Tal informação nos faz pensar se as crianças com TEA podem responder, mesmo que inadequadamente. Mas é analisando a função dessa resposta é que conseguimos compreender melhor a falha, que se encontra de maneira basal na socialização. Podemos fazer essa associação também com a análise de linguagem pragmática, em que olharmos categoricamente para um aspecto ou uma habilidade, muitas vezes não se configura ou classifica como uma falha. Mas ao analisarmos o uso dessa habilidade, os contextos em que ocorrem essa habilidade, percebemos com mais clareza a deficiência no âmbito social já que lançamos luz também à sua função.

Conforme indicado nos resultados, ao associarmos a variável “verbais e não verbais”, as crianças *não verbais* apresentaram valores médios e medianos superiores para os escores analisados (p<0.050), indicando maior dificuldade ou déficit. Mesmo o ROLPP não dependendo da oralidade da criança para ser aplicado, ele pode indicar um alerta quanto ao atraso no desenvolvimento de linguagem, não apenas focado na ausência e presença de fala, mas também associando à linguagem pragmática. Em crianças na primeiríssima infância é ainda mais difícil identificar o que é um déficit primário de comunicação social e quais os sintomas que são consequência de déficits no desenvolvimento da linguagem ou de outras áreas como o desenvolvimento cognitivo, social, emocional e motor. No desenvolvimento inicial, estas áreas são interdependentes e em constante evolução, aumentando o desafio do diagnóstico precoce^(49,50)^.

Quanto à eficiência do questionário, os dados mostraram que o ROLPP foi capaz de identificar déficits em quatro áreas de desenvolvimento essenciais ao desenvolvimento da linguagem: responsividade, contato visual e atenção compartilhada, imitação e jogo simbólico. Esses resultados eram esperados, uma vez que os prejuízos na comunicação social são uma das principais características do TEA. Ao comparar as médias dos dois grupos notou-se que houve diferença significativa comparando as quatro áreas (valores de p: Responsividade 0,009; CO&AC <0,001; Imitação 0,01; Jogo Simbólico 0,01). Isso mostra que crianças com diagnóstico confirmado tendem a ter maior pontuação no ROLPP em geral (p-valor: <0,001), evidenciando os impactos da alteração de linguagem pragmática em crianças com TEA mesmo em idades jovens.

## CONCLUSÃO

Esses resultados preliminares do ROLPP indicam a possibilidade de identificação de sinais de transtornos ou dificuldades pragmáticas antes mesmo dos 36 meses para uso de profissionais fonoaudiólogos sem muita experiência ou contato com transtornos do desenvolvimento como TEA visando aprimorar a triagem e encaminhamento para avaliação especializada de forma precoce.

O ROLPP se mostrou um recurso efetivo de baixo custo, fácil de implementar na prática clínica. No entanto, faz-se necessário mais estudos com o seu uso para melhor compreensão dos seus benefícios e desafios. Vale também considerar a relevância de um modelo de observação sistemática da comunicação espontânea da criança com um adulto familiar, evitando a interferência de fatores motivacionais, de familiaridade, linguísticos e culturais. Visto que a avaliação e a intervenção precoce é um dos fatores mais impactantes no prognóstico de crianças com alterações de linguagem, o uso de instrumentos que facilitam e encurtam esse processo inicial é fundamental.
